# Ultrasonographic placental thickness versus fetal outcome: A prospective study in Southern India

**DOI:** 10.22088/cjim.12.4.562

**Published:** 2021

**Authors:** Gauri Raghunath Shinde, Nitin Kshirsagar, Manisha Laddad, Vaishnavi Shivade

**Affiliations:** 1 Department of Obstetrics and Gynecology, Krishna Institute of Medical Sciences (Deemed to be) University, Malkapur, Maharashtra, India

**Keywords:** Gestational diabetes, Gestational weight gain, Fetal growth, Placenta previa

## Abstract

**Background:**

Variations in placental thickness are associated with increased perinatal morbidity and mortality. However, only very few studies have been established on the correlation between placental thickness with birth outcomes. This study correlated placental thickness in 2^nd^ and 3^rd^ trimesters with neonatal outcome, maternal weight gain, and body mass index (BMI).

**Methods:**

A total of 116 patients aged between 20 to 50 years with singleton pregnancy and regular menstrual history (and sure about their last menstrual period) were included. Placental thickness was measured at 24 and 36 weeks by ultrasound and was divided into three groups: Group A (normal placenta), Group B (thin placenta), and Group C (thick placenta); and correlated with neonatal outcome, maternal weight gain, and BMI.

**Results:**

Out of the 116 pregnant women, 55 (47.4%) were primigravida and 61 (52.6%) were multigravida. Six patients (3.6%) delivered pre-term before 36 weeks. In the 2^nd^ and 3^rd^ trimesters, most cases had normal placental thickness (Group A; 93.1% and 92.7%), followed by thin placenta (Group B; 5.2% and 7.3%) and thick placenta (Group C; 1.7% and 0), respectively. Two patients with thin placenta had neonatal death. A significant positive correlation was found between birth weight and placental thickness (at 24 weeks; 0.516^r^, *P*<0.00001 and at 36 weeks; 0.669^r^, P<0.00001) and maternal weight gain and birth weight (0.563^r^, P<0.00001).

**Conclusion:**

Placental thickness on ultrasonography demonstrated well the correlation between birth weight in 2^nd^ and 3^rd^ trimesters and increased incidence of antenatal and postpartum complications resulting from thin placenta.

Placenta is an important fetal organ with metabolic, immunological, endocrinal, respiratory, and nutritional functions. It also plays a vital role in protecting the fetus by acting as a barrier against infections and toxic substances. Normal placental structure and function are required for normal fetal growth and development. In term pregnancy, the weight of the placenta is about one-fifth of the weight of the fetus. Change in maternal metabolism affects the placental function and its morphology which ultimately affects birth weight at delivery. Maternal weight gain during pregnancy directly affects the growing fetus and indirectly the adult health outcome (1, 2). With the invention of ultrasonography and its newer advancements, it is now possible to do Doppler imaging of the placenta and study its appearance, uteroplacental circulation, and its variability in complicated pregnancies (3). Placental thickness has been noted to increase as pregnancy advances. Its thickness at the cord insertion site was found to have a linear relation with the gestational age.

Also, it was found that variations in placental thickness were associated with increased perinatal morbidity and mortality (4). Low birth weight (LBW) is an extensively established risk factor for long-term effects, especially metabolic and cardiovascular disorders (4). Recently, researchers have identified many determinants of abnormal (both low and high) neonatal birth weight (5, 6, 7). Thick placenta is observed in Rh-ve pregnancy, intrauterine infections, gestational diabetes, and fetal hydrops, whereas thin placenta is observed in preeclampsia, chorioamnionitis, and intrauterine growth restriction (IUGR) (5). Few studies have demonstrated the role of placental thickness in predicting the fetal outcome and fewer studies have established an association between placental thickness at different gestational ages and birth weights (1, 2). A study conducted in Iranians reported a weak positive correlation between placental thickness and fetal weight and birth weight (8). However, the role of normal, thin, and thick placenta in determining the fetal outcome is still inconclusive. In addition, most of the established studies were retrospective in design (9, 10, 11). Hence, there is a dearth of prospective and follow-up studies to establish an association between placental thickness and neonatal outcome. Thus, this study was intended to correlate placental thickness in 2^nd^ and 3^rd^ trimesters with neonatal outcome, maternal weight gain, and body mass index (BMI).

## Methods

After obtaining ethical clearance (KIMSDU/IEC-307/032/26/03/2018), this prospective, observational study was carried out for one year between April 2018 and March 2019 in the Department of Obstetrics and Gynecology at a tertiary care teaching hospital, Karad, Maharashtra, India. Sample size was calculated using Cohen’s d effect size with the expected correlation coefficient (r) of 0.741 for an effect size (r=0.3. medium) at a significance level 95% and power 90%. A minimum sample of 112 participants was required, but we recruited 116 patients so that we had an adequate number if there were any dropouts from the study.

A total of 116 antenatal women, aged 20 to 50 years, with singleton pregnancy, regular menstrual history (and sure about their last menstrual period), and no history of oral contraceptive usage prior to conception were included in the study, after obtaining written informed consent. Women with pregnancy risk factors (viz., hypertension, diabetes, chronic renal disease, and sickle cell anemia), fetal congenital abnormalities, placental anomalies, placental abruption, placenta previa, and multiple pregnancies were exempted from the study.

Each patient underwent ultrasonography (Philips HDI 4000 using a curvilinear transducer-3.5MHz) for placental thickness measurement at 24 weeks and 36 weeks during 2^nd^ and 3^rd^ trimester, respectively. Placenta was localized in a longitudinal section. The placental thickness was measured at the level of umbilical cord insertion in longitudinal direction from the lateral chorionic plate to the cord insertion excluding the retro placental area; the maximum thickness was noted in the cross section. All patients were in the supine position with a full urinary bladder while they underwent the ultrasonography and observed for any variation in the placental thickness till delivery (12). For the convenience of our study, the placental thickness was calculated in percentiles. Based on the placental thickness, all the pregnant women were categorized into three groups: Group A (normal placenta; placental thickness between 10^th^ and 95^th^ percentile), Group B (thin placenta; placental thickness <10^th^ percentile or < mean - 2SD), and Group C (thick placenta; placental thickness >95^th^ percentile or > mean + 2SD) (12). The pregnant women in Groups B and C were monitored closely and followed-up till delivery to observe for any signs of IUGR, preterm labor, maternal pregnancy-induced hypertension (PIH), gestational diabetes mellitus (GDM), and abortion. Post-delivery neonatal birth weight, Apgar score, need of NICU admission, and mode of delivery were recorded.

Statistical analysis was done using R Version 3.6.0. software. Normality of the data was determined using the Shapiro-Wilk test. The continuous variables with normal distribution were presented as mean±standard deviation, whereas the categorical variables were presented as frequencies and percentages. The correlation was determined for placental thickness with neonatal birth weight, BMI, and maternal weight gain by Pearson’s correlation analysis test. A *p-*value of <0.05 was considered statistically significant at 95% confidence interval.

## Results

Out of 116 pregnant women, 55 (47.4%) were primigravida and 61 (52.6%) were multigravida. The mean age and BMI of all pregnant women were 26.2±4.06 years and 20.8±1.46 kg/m^2^, respectively. Majority of them were in the age group of 23-28 years. Twenty-two patients had low BMI (18-20 kg/m^2^). The mean placental thickness at 24 weeks and 36 weeks during 2^nd^ and 3^rd^ trimester was 24.05±0.21 and 35.31±0.5 mm, respectively. Six (3.6%) patients delivered preterm (before 36 weeks) and, therefore, could not undergo the third trimester ultrasound for placental thickness. Consequently, only 110 pregnant women were considered for the measurement of placental thickness during the 3^rd^ trimester. A mean placental thickness of ≤21.7 mm and ≤29.9 mm was considered as thin placenta in 2^nd^ and 3^rd^ trimesters, respectively. Placental thickness ≥26.4 mm and ≥40.7 mm was considered as thick placenta in 2^nd^ and 3^rd^ trimesters, respectively. Table 1 represents the distribution of pregnant women with normal (Group A), thin (Group B), and thick placenta (Group C) in 2^nd^ and 3^rd^ trimesters.

Pregnant women with thin placenta at 24 weeks (06; 5.2%) delivered very low birth weight (LBW) neonates (<2 kg) who were shifted to the NICU. Two among these six pregnant women had preterm delivery and the two neonates died in the NICU, probably due to preterm birth and/or acute respiratory distress syndrome (ARDS). All the pregnant women with thick placenta at 24 weeks (02; 1.7%) delivered high birth weight neonates (≥3 kg); one of these two pregnant women had glucose intolerance with a maternal weight gain of 13 kg. Table 2 represents the antenatal and postpartum complications among pregnant women with normal (Group A), thin (Group B), and thick placenta (Group C). In Group C, two women with polyhydramnios had postpartum hemorrhage (PPH) and were treated conservatively with uterotonics. Table 3 represents the mode of deliveries with regard to the gestational age at delivery (preterm, full-term, and post-dated).

The mean birth weight was 2.63±1.2 kg. Eight (6.9%) neonates had LBW and two among them died, whereas the remaining 106 (93.10%) babies were born with normal birth weight. Two pregnant women with low BMI had thin placenta at 2^nd^ trimester and two at 3^rd^ trimester. The mean maternal weight gain during pregnancy was 10.4 ± 0.8 kg. Six pregnant women had maternal weight loss (5-6 kg) during pregnancy and delivered LBW neonates. Table 4 represents the correlation between maternal and neonatal variables. A negative and positive linear correlation was observed for maternal BMI (- 0.061^r^ and *P*=0.516) versus birth weight and maternal weight gain versus birth weight (0.563^r^ and *P*<0.00001), respectively.

**Table 1:** USG placental thickness in 2nd and 3rd trimester in the study groups

Groups	At 24 weeks during 2^nd ^trimester (N=116); n (%)	At 36 weeks during 3^rd^ trimester (N=110); n (%)
**A**	108 (93.1)	102 (92.7)
**B**	6 (5.2)	8 (7.3)
**C**	2 (1.7)	0

**Table 2:** Antenatal and postpartum complications

Complication	Group A (N)	Group B (N)	Group C (N)
**Severe PIH**	02	04	0
**IUGR**	04	06	0
**Eclampsia**	01	01	0
**Oligohydramnios**	01	02	01
**Polyhydramnios**	02	0	02
**Glucose intolerance**	02	0	01
**GDM**	0	0	01
**Preterm delivery**	0	06	0
**Neonatal death**	0	02	0
**NICU admission**	02	06	0
**Poor APGAR (<4 at 1 min)**	02	06	0

GDM= Gestational diabetes mellitus,

NICU = Neonatal intensive care unit, IUGR= Intrauterine growth restriction,

PIH= Pregnancy induced hypertension

**Table 3:** Comparison of gestational age at delivery and mode of delivery

Mode of delivery		Preterm (N=06)	Full-term (N=70)	Post-dated (N=40)
**Vaginal delivery**		05	35	20
**Instrumental delivery**		0	04	05
**VBAC**		0	02	0
**LSCS**	Elective	0	18	0
	Emergency	01	10	151

LSCS = Lower segment cesarean section, VBAC = Vaginal delivery after cesarean section

**Table 4:** Test results for correlation of placental thickness with maternal BMI and birth weight

Variables	r	*P* value	Significance
**Placental thickness (at 24 weeks) versus birth weight**	0.516	<0.00001	Positive linear correlation (S)
**Placental thickness (at 36 weeks) versus birth weight**	0.669	<0.00001	Positive linear correlation (S)
**Placental thickness (at 24 weeks) versus BMI**	- 0.057	0.543	Negative linear correlation (NS)
**Placental thickness (at 36 weeks) versus BMI**	- 0.136	0.153	Negative linear correlation (NS)
**Placental thickness (at 24 weeks) versus maternal weight gain**	0.413	<0.00001	Positive linear correlation (S)
**Placental thickness (at 36 weeks) versus maternal weight gain**	0.564	<0.00001	Positive linear correlation (S)

BMI= Body mass index, P= Fitting generalized linear model, r= Pearson’s correlation coefficient, S= Significant, NS= Nonsignificant

## Discussion

Normal placental structure and function are essential for normal fetal growth and development. Adverse neonatal outcomes and fetal growth (placental efficiency) differ significantly based on geographical and ethnic backgrounds. Thus, in this study, placental thickness was used to assess the neonatal outcome as many researchers have extensively studied the association between placental thickness and adverse neonatal outcomes (1, 2, 11, 13, 14). A relatively low incidence of thin placenta (12.5%) was observed compared to thick placenta (2.7%) in our study. Audette et al. conducted a study on 829 nulliparous pregnant women and reported a high incidence of thin placenta (24.2%) in South Asian pregnant women (15). These contradictory findings could be due to a smaller number of samples in our study. Thin placenta can be due to IUGR, preeclampsia, and chorioamnionitis (11, 13). 

A prospective study conducted by Afrakhteh et al. reported a positive linear correlation between placental thickness and fetal age (8). Mathai et al. in 2013 evaluated the correlation of placental thickness in 498 patients with gestational age and fetal outcome by dividing them into two groups—Group A (fetal weight <2500 g) and Group B (fetal weight >2500 g). They found a moderate positive correlation between ultrasonographic gestational age and placental thickness in both groups. They also concluded that mean placental thickness in Group A is relatively lower compared to Group B (16). 

In Philadelphia, Schwartz et al. conducted a study on women aged between 18 and 24 years with singleton pregnancies (n=1909) and reported that preterm neonates had significantly smaller mean placental thickness (1). Accordingly, a moderate positive linear correlation was found between placental thickness and birth weight at 24 weeks (0.516^r^) and 36 weeks (0.669^r^), whereas Kashika et al. reported a strong positive correlation at 32 (0.55^r^) and 36 (0.74^r^) weeks of gestational age (12). In concordance with our study results, Afrakhteh et al. conducted a prospective study involving 250 singleton pregnancies and reported a positive correlation between placental thickness and birth weight in 2^nd^ and 3^rd^ trimesters (8). However, they concluded that change in placental thickness could not predict LBW (8).

In the present study, an increased incidence of antenatal, intrapartum, and postpartum development of multiple complications (viz, PIH, IUGR, preterm delivery, oligohydramnios, LBW neonates, NICU admission, poor Apgar score (<4 at 1 min), and a need for emergency LSCS) was observed in pregnant women with thin placenta, whereas increased incidence of polyhydramnios was found with thick placenta. In contrast, Kashika et al. found increased incidence of poor Apgar scores, NICU admissions, and LBW neonates with thick placenta (12). 

The incidence of the perinatal mortality and the fetal anomalies were greater in the subjects with thick placentas. Ahmed et al. who conducted a study in pregnant Sudanese women (n=53) in 2^nd^ and 3^rd^ trimesters observed higher incidence of IUGR with thin placenta (<25 mm) at 36 weeks of gestational age and concluded that thin placenta could be a predictor of IUGR, whereas thick placenta (>45 mm) could be a predictor of GDM, PIH, and hydropsfetalis (17). Accordingly, an incidence of polyhydramnios, glucose intolerance, and GDM was observed with only thick placenta (≥26.4 and ≥40.7 mm at 24 and 36 weeks of gestational age, respectively) in the present study. Subnormal placental thickness may be an earliest indicator of IUGR, which can be treated if it is diagnosed at the earliest. An enlarged placenta (placentomegaly) is suspected if the PT is > 40 mm at term and if it is associated with GDM, intra-uterine infections, hydropsfetalis, anemia, and α- thalassemia type. So, an increased placental thickness for that gestational age should raise a suspicion about the possible disease conditions (18).

In the US, a study conducted on 24,000 placentas explored the association between placental measures and maternal characteristics and reported that a 36.5% variation in the fetal weight is totally based on the placental weight, whereas maternal characteristics (viz, age, BMI, parity, ethnicity, cigarette use, and socio-economic status) accounted for 13.9% variation in the fetal weight (19). 

In the present study, a moderate positive correlation (0.563^r^) was found between the birth weight and maternal weight gain, whereas a negative correlation (0.061^r^) was observed between birth weight and BMI. Maternal weight gain and BMI (pre pregnancy) were identified as indicators of placental hypertrophy in all the three dimensions of its growth. Currently, available literature demonstrates that effects of maternal weight gain and BMI on fetal growth and birth weight at least partially affects the placental growth and its properties (20).

Our study had few major limitations. First, a small number of patients, which would be the reason for lower incidence of abnormal placental thickness. Second, we did not consider the nutritional and socio-economic status of the included women while assessing the neonatal outcomes in correlation with placental measures. Further studies are required to evaluate the impact of lifestyle habits and nutritional and socio-economic status on birth events.

The placental thickness on ultrasonography demonstrated well the correlation between birth weight in 2^nd^ and 3^rd^ trimesters and increased incidence of antenatal and postpartum complications found with thin placenta. Thus, placental thickness could be a good predictor in the early detection of fetuses that are at increased risk.
